# Predictors of one and two years' mortality in patients with colon cancer: A prospective cohort study

**DOI:** 10.1371/journal.pone.0199894

**Published:** 2018-06-28

**Authors:** José M. Quintana, Ane Antón-Ladislao, Nerea González, Santiago Lázaro, Marisa Baré, Nerea Fernández-de-Larrea, Maximino Redondo, Eduardo Briones, Antonio Escobar, Cristina Sarasqueta, Susana García-Gutierrez, Inmaculada Aróstegui, for the REDISSEC-CARESS/CCR group

**Affiliations:** 1 Unidad de Investigación, Hospital Galdakao-Usansolo, Galdakao, Bizkaia, Spain; 2 Red de Investigación en Servicios Sanitarios y Enfermedades Crónicas (REDISSEC), Galdakao, Bizkaia, Spain; 3 Servicio de Cirugía General, Hospital Galdakao-Usansolo, Galdakao, Bizkaia, Spain; 4 Unidad de Epidemiología Clínica, Corporació Parc Taulí, Sabadell, Spain; 5 Centro Nacional de Epidemiología, Instituto de Salud Carlos III, Madrid, Spain; 6 CIBER Epidemiología y Salud Pública (CIBERESP), Madrid, Spain; 7 Unidad de Investigación, Hospital Costa del Sol, Málaga, Spain; 8 UDG Salud Pública, Distrito AP Sevilla, Sevilla, Spain; 9 Unidad de Investigación, Hospital Universitario Basurto, Bilbao, Bizkaia, Spain; 10 Unidad de Investigación, Hospital Universitario Donostia/Biodonostia, Donostia-San Sebastian, Gipuzkoa, Spain; 11 Departamento de Matemática Aplicada, Estadística e Investigación Operativa, UPV/ EHU, BCAM-Basque Center for Applied Mathematics, Leioa, Bizkaia, Spain; Okayama Daigaku, JAPAN

## Abstract

**Background:**

Tools to aid in the prognosis assessment of colon cancer patients in terms of risk of mortality are needed. Goals of this study are to develop and validate clinical prediction rules for 1- and 2-year mortality in these patients.

**Methods:**

This is a prospective cohort study of patients diagnosed with colon cancer who underwent surgery at 22 hospitals. The main outcomes were mortality at 1 and 2 years after surgery. Background, clinical parameters, and diagnostic tests findings were evaluated as possible predictors. Multivariable multilevel logistic regression and survival models were used in the analyses to create the clinical prediction rules. Models developed in the derivation sample were validated in another sample of the study.

**Results:**

American Society of Anesthesiologists Physical Status Classification System **(**ASA), Charlson comorbidity index (> = 4), age (>75 years), residual tumor (R2), TNM stage IV and log of lymph nodes ratio (> = -0.53) were predictors of 1-year mortality (C-index (95% CI): 0.865 (0.792–0.938)). Adjuvant chemotherapy was an additional predictor. Again ASA, Charlson Index (> = 4), age (>75 years), log of lymph nodes ratio (> = -0.53), TNM, and residual tumor were predictors of 2-year mortality (C-index:0.821 (0.766–0.876). Chemotherapy was also an additional predictor.

**Conclusions:**

These clinical prediction rules show very good predictive abilities of one and two years survival and provide clinicians and patients with an easy and quick-to-use decision tool for use in the clinical decision process while the patient is still in the index admission.

## Introduction

Colon cancer is one of the most frequently diagnosed cancers and is increasing in prevalence in some countries, partly owing to better screening and diagnosis strategies. Despite this increased prevalence, survival expectancy is improving, owing to earlier detection and better tools for treatment.[1,2] Most patients will undergo surgery and some will also receive chemotherapy. However, mortality remains high and, so, a main outcome of interest. Therefore, there is concern about the prognosis of these patients in terms of identifying predictors of mortality based on patient characteristics.[3] For this assessment, prediction models, decision rules, and risk scores are statistical tools intended to classify patients and guide clinicians in their everyday decision-making. These tools consist of a combination of multiple predictors, such as patient characteristics, diagnosis, and evolution, to estimate the probability of a certain outcome.[4,5] Different researchers have also developed various prediction rules for colon cancer patients, mainly to predict survival after surgery or very shortly afterwards.[6,7] Five-year survival prediction has been the outcome of some other studies.[8–10] There are fewer studies trying to predict survival at a longer period of time, such as 1–2 years.

The goal of this study was to develop clinical prediction rules for 1- and 2-year mortality in colon cancer patients who underwent surgery with the purpose of helping to personalize treatment and assist in the follow-up of these patients.

## Methods

This prospective cohort study included patients drawn from 22 hospitals belonging to the Spanish National Health Service, which covers the majority of the Spanish population. All covered residents have free access to their primary care physician and the Emergency Department (ED) of the hospitals. All of the hospitals have similar technological and human resources.

A description of the study protocol is detailed elsewhere.[11] In summary, patients diagnosed with colon cancer presenting at any of these hospitals to undergo surgery between June 2010 and December 2012 were informed of the goals of the study and invited to participate. In order to take part in the study, a patient had to provide a signed informed consent. All information was kept confidential. The Institutional Review Board of the Basque Country approved this project.

Patients were eligible for the study if they were included in the surgical waiting list of one of the participating hospitals with a diagnosis of surgically resectable colon cancer. Colon cancer diagnosis was based on anatomopathological diagnosis after a biopsy by colonoscopy. Inclusion criteria were: 1) having been diagnosed with colon cancer(up to 15 cm above the anal margin), 2) initial application of curative and/or palliative surgery for treatment, and 3) signing the informed consent to participate in the study.[12] Exclusion criteria were: 1) colon carcinoma *in situ*, 2) unresectable tumor, 3) severe mental or physical conditions that precluded the patient from responding to questionnaires, 4) terminal disease, 5) inability to respond to questionnaires from any cause, and 6) did not give consent to participate in the study. We estimated the sample size needs of the study based on current recommendations for clinical prediction rules based on the expected rates of the outcome of interest (mortality at 1 or 2 years).[5,13]

Data collected upon hospital admission included information on sociodemographic factors, clinical data (including information about onset of symptoms, habits, personal and family background, comorbidities including those of the Charlson Comorbidity Index,[14] diagnostic tests, and pre-intervention treatments), preoperative data (including American Society of Anesthesiologists Physical Status Classification System (ASA) stage,[15] analytical data, tumor markers, and diagnostic tests), outpatient anesthesia data from the surgical intervention, pathological data, and data related to the remainder of the hospital stay (including the presence of complications, need for reoperation, or death). Subsequently, data were collected up to 30 days after surgery (analytical data, diagnostic tests, presence of complications, readmissions, reoperation, or death). Finally, patient information was collected through the first and second postoperative year, including radiation therapy, chemotherapy (treatment schedule, cycles, complications of treatment, and supportive care), laboratory results and diagnostic tests, presence of complications, tumor recurrence, readmission or reoperation, and death.

### Outcome measures

The primary outcomes were mortality at 1 and 2 years after the patient was first admitted to the hospital (index admission). Vital status was established by reviewing medical records and examining the hospital database and public death registries. Deaths were considered confirmed if the name, sex, and date of birth on the record matched those of the participant.

### Statistical analyses

The unit of analysis was a patient with a diagnosis of colon cancer who underwent operation in one of the participating hospitals. The sample was randomly divided in two subsamples (derivation and validation), each with half of the total population. Randomization of patients was performed automatically by a statistical program. To ease the interpretation of all the models, as well as, the punctuation of the scores derived from the models, all continuous variables were categorized. In the case of the age and Charlson comorbidity index, the optimal categorization of both was performed following the methodology developed by Barrios I et al [16]. In the case of Log Lymph ratio, categorization of the continuous variable was done based on the publication of Persiani R et al [17]. Descriptive statistics for both samples included frequencies and percentages for categorical data and means and standard deviations for continuous variables.

Univariable analysis was first performed in the derivation sample to identify risk factors related to mortality up to 1 and 2 years, using univariable Cox proportional-hazards regression models. Variables that were significant at a level of 0.20 were considered as potential independent variables for the multivariable analysis. Multivariable Cox proportional-hazards regression models were developed for mortality up to 1 and 2 years. Final predictive factors in multivariable analysis were those with a significance level of 0.05. Final models were also adjusted by the treating hospital to see if that affected the results and evaluated in the validation sample. As an internal validation of the models, 200 bootstrap samples with replacement were drawn from the original sample, and using for the selection of the variables stepwise method, 200 different models were developed in each sample. In order to compare the results of the 200 models with the model fitted in the original sample, the times that different predictors were predictors in the final models of each sample were summarized. Additionally, presence of collinearity was evaluated for some variables. Indeed, we developed two severity risk scores (for mortality up to 1 and 2 years) by assigning a weight to each risk factor category based on the β parameter from the multivariable Cox proportional-hazards regression. Then we added the weights of each of the risk factors presented by a patient with a higher score corresponding to a higher likelihood of mortality. Considering the optimal classifier points, three severity categories were created in each score.[16]

The predictive accuracy of each model was determined by the C-index. The predictive capacity of the risk groups derived from the models was also evaluated and validated in both samples by calculating the area under the receiver operating characteristic curve (AUC) and the calibration of the models was tested by Hosmer-Lemeshow test. All effects were considered significant at p < 0.05. All statistical analyses were performed using SAS 9.4 software (SAS Institute Inc., Cary, NC, USA).

## Results

**Fig 1:**
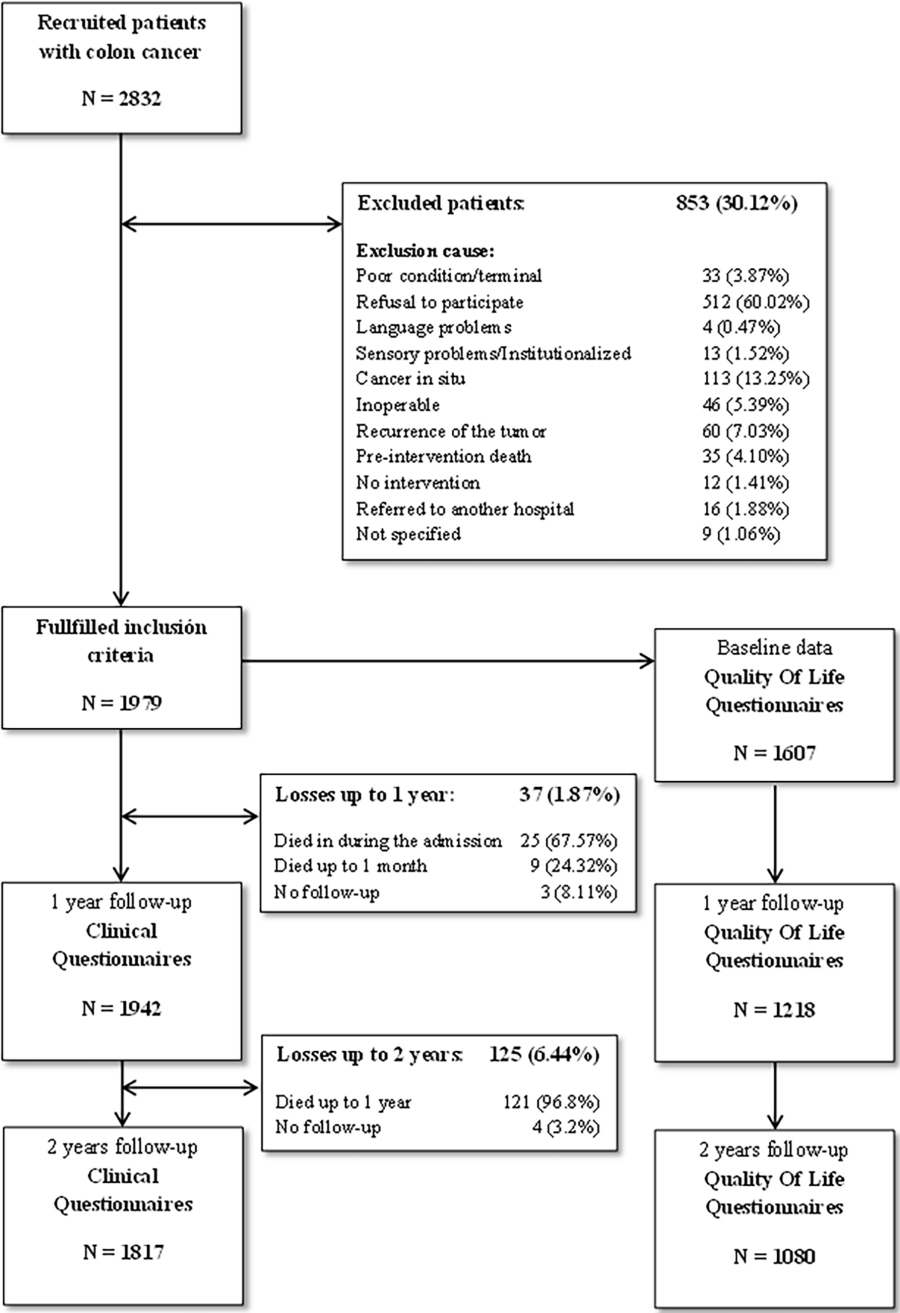
Flow-chart of the recruitment and follow-up process.

We initially contacted 2832 patients. Of these patients, 1979 fulfilled the selection criteria, with 1942 patients having full information at 1 year and 1817 at 2 years of follow-up as displayed in “Fig 1”.

Table 1 shows the descriptive of the derivation, validation and whole samples. Table 2 shows the different chemotherapy strategies employed in our patients. Univariable analysis in the derivation sample of the relationship of different sociodemographic and clinically relevant variables after 30 days from surgery with mortality at 1 (6.58% mortality rate) and 2 years (6.59% mortality rate) is displayed in Table 3.

**Table 1:** Descriptive sociodemographic and clinical statistics of all samples of the study.

	N (%)	Sample		p-value
		Derivation N (%)	Validation N (%)	
**Total**	1945	972 (49.97)	973 (50.03)	
Gender (Male)	1205 (61.95)	611 (62.86)	594 (61.05)	0.4106
Age				0.3674
• **≤75**	1339 (68.95)	678 (69.90)	661 (68.00)	
• **>75**	603 (31.05)	292 (30.10)	311 (32.00)	
Haemoglobin at baseline*	12.33 (2.15)	12.38 (2.12)	12.28 (2.17)	0.3834
Charlson index				0.8181
• **<4**	1511 (77.69)	753 (77.47)	758 (77.90)	
• **≥4**	434 (22.31)	219 (22.53)	215 (22.10)	
ASA				0.4318
• **I,II,III**	1817 (95.93)	908 (95.58)	909 (96.29)	
• **IV**	77 (4.07)	42 (4.42)	35 (3.71)	
Type of surgery				0.6986
• **Laparoscopy**	1138 (59.02)	566 (58.59)	572 (59.46)	
• **Open Surgery**	790 (40.98)	400 (41.41)	390 (40.54)	
Organ invasión				0.2322
• **0**	1741 (89.51)	860 (88.48)	881 (90.54)	
• **1**	167 (8.59)	94 (9.67)	73 (7.50)	
• **>1**	37 (1.90)	18 (1.85)	19 (1.95)	
Result of the surgery				0.4683
• **R0**	1741 (93.10)	862 (92.39)	879 (93.81)	
• **R1**	72 (3.85)	39 (4.18)	33 (3.52)	
• **R2**	57 (3.05)	32 (3.43)	25 (2.67)	
Log Lymph nodes ratio				0.5683
• **≤-1.36**	1528 (81.62)	754 (80.99)	774 (82.25)	
• **-1.36< ≤-0.53**	184 (9.83)	91 (9.77)	93 (9.88)	
• **-0.53<**	160 (8.55)	86 (9.24)	74 (7.86)	
Length of stay†	9 [7–14]	9 [7–14]	9 [7–13]	0.3787
Adjuvant Chemotherapy and pTNM				0.3078
• **No chemo and pTNM 0, I, II**	842 (43.88)	399 (41.74)	443 (46.00)	
• **Yes chemo and pTNM 0, I, II**	238 (12.40)	127 (13.28)	111 (11.53)	
• **No chemo and pTNM III**	157 (8.18)	87 (9.10)	70 (7.27)	
• **Yes chemo and pTNM III**	501 (26.11)	247 (25.84)	254 (26.38)	
• **No chemo and pTNM IV**	35 (1.82)	19 (1.99)	16 (1.66)	
• **Yes chemo and pTNM IV**	146 (7.61)	77 (8.05)	69 (7.17)	
*Outcomes*				
Mortality up to 1 year	121 (6.22)	62 (6.38)	59 (6.06)	0.7738
Mortality up to 2 years	236 (12.13)	118 (12.14)	118 (12.13)	0.9933

N: Frequency, %: Percentage

*Results shown as mean (standard deviation).

†Results shown as median [25^th^ percentil– 75^th^ percentil].

R-stage of the operation. Residual tumor (R) classification: R0, no residual tumor; R1, microscopic residual tumor; R2, macroscopic residual tumor

**Table 2:** Descriptive statistics of the adjuvant chemotherapy and molecular targeted agents employed in each sample.

	N (%)	Sample		p-valor
		Derivation N (%)	Validation N (%)	
**Total**	1945	972 (49.97)	973 (50.03)	
Adjuvant chemotherapy	892 (46.19)	457 (47.46)	435 (44.94)	0.2672
• **Folfox (Fluorouracil, Oxaliplatin)**	239 (12.38)	128 (13.29)	111 (11.47)	0.2234
• **Folfiri (Fluorouracil, Irinotecan, folinic acid)**	50 (2.59)	25 (2.60)	25 (2.58)	0.9852
• **Capeox/Xelox (Capecitabine, Oxaliplatin)**	429 (22.22)	226 (23.47)	203 (20.97)	0.1869
• **Fluorouracil**	8 (0.41)	2 (0.21)	6 (0.62)	0.2881
• **Fluoruracil-Leucovorín**	30 (1.55)	17 (1.77)	13 (1.34)	0.4531
• **Monotherapy**	169 (8.75)	69 (7.17)	100 (10.33)	0.0139
Molecular targeted agents	107 (5.61)	59 (6.20)	48 (5.02)	0.2593

N: Frequency, %: Percentage

**Table 3:** Univariable analysis in the derivation sample for mortality up to 1 and 2 years.

	**Univariable analysis**			
	Mortality up to 1 year HR (95% CI)	p-value	Mortality up to 2 years HR (95% CI)	p-value
**Total‡**	64 (6.58)		124 (12.76)	
Age				
• **≤75**	Ref.		Ref.	
• **>75**	2.435 (1.473–4.026)	0.0005	2.185 (1.520–3.142)	<0.0001
Haemoglobin at baseline*	0.985 (0.953–1.018)	0.3627	0.990 (0.973–1.008)	0.2646
Charlson index				
• **<4**	Ref.		Ref.	
• **≥4**	2.199 (1.307–3.701)	0.0030	1.729 (1.174–2.546)	0.0056
ASA				
• **I,II,III**	Ref.		Ref.	
• **IV**	4.889 (2.404–9.942)	<0.0001	4.187 (2.393–7.326)	<0.0001
Type of surgery				
• **Laparoscopy**	Ref.		Ref.	
• **Open surgery**	1.789 (1.064–3.008)	0.0284	1.590 (1.100–2.297)	0.0135
Organ invasion				
• **0**	Ref.		Ref.	
• **1**	2.747 (1.433–5.268)	0.0023	2.939 (1.879–4.598)	<0.0001
• **>1**	13.196 (6.413–27.154)	<0.0001	8.411 (4.366–16.203)	<0.0001
Tumor sidedness				
• **Right**	1.529 (0.921–2.536)	0.1005	1.583 (1.101–2.275)	0.0131
• **Left**	Ref.		Ref.	
Histology				
• **Adenocarcinoma**	Ref.		Ref.	
• **Others**	2.357 (1.344–4.133)	0.0028	1.926 (1.259–2.945)	0.0025
Result of the surgery				
• **R0**	Ref.		Ref.	
• **R1**	3.618 (1.415–9.250)	0.0073	2.924 (1.467–5.827)	0.0023
• **R2**	15.624 (8.896–27.442)	<0.0001	11.562 (7.399–18.070)	<0.0001
Log Lymph nodes ratio				
• **≤-1.36**	Ref.		Ref.	
• **-1.36< ≤-0.53**	4.106 (1.933–8.719)	0.0002	3.196 (1.918–5.325)	<0.0001
• **-0.53<**	10.251 (5.647–18.607)	<0.0001	5.997 (3.865–9.307)	<0.0001
Length of stay†	1.015 (1.007–1.024)	0.0005	1.013 (1.006–1.020)	0.0002
Adjuvant Chemotherapy and pTNM				
• **No chemo and pTNM 0, I, II**	Ref.		Ref.	
• **Yes chemo and pTNM 0, I, II**	1.132 (0.235–5.447)	0.8774	0.789 (0.270–2.307)	0.6646
• **No chemo and pTNM III**	10.688 (4.357–26.217)	<0.0001	7.071 (3.947–12.670)	<0.0001
• **Yes chemo and pTNM III**	2.016 (0.750–5.417)	0.1644	1.934 (1.068–3.501)	0.0294
• **No chemo and pTNM IV**	24.276 (7.702–76.516)	<0.0001	14.057 (5.940–33.267)	<0.0001
• **Yes chemo and pTNM IV**	10.756 (4.251–27.212)	<0.0001	9.491 (5.328–16.906)	<0.0001

Excluded patient who died throughout the admission or during the first 30 days after admission

*Results shown as mean (standard deviation).

†Results shown as median [25^th^ percentil– 75^th^ percentil].

‡Frequency (percentage) of patients who die up to 1 year or up to 2 years.

† HR: Hazard Ratio. CI: Confidence Interval

*†HR estimated for a unit increase.

R-stage of the operation. Residual tumor (R) classification: R0, no residual tumor; R1, microscopic residual tumor; R2, macroscopic residual tumor.

Table 4 shows, based on the multivariable analysis, predictors of 1-year mortality were the ASA IV, a score ≥ 4 in the Charlson comorbidity index (CCI), age older than 75 years, TNM stage IV, lymph nodes ratio (>-0.53) and the residual tumor classification (R2 vs. R0-R1 categories). All the significant variables in the derivation sample were also significant in the validation sample. The C-index showed a good discrimination for both samples (0.865 (0.792–0.938) and 0.808 (0.734–0.882), in the derivation and validation samples, respectively). When including the treating hospital in the model, the C-index rose to 0.881 (0.807–0.955) in the derivation sample and 0.852 (0.776–0.928) in the validation sample. Since the use of adjuvant chemotherapy after 30 days of surgery has a role in survival, this variable was introduced as an interaction term with TNM stage. C-indexes were 0.885 (0.797–0.973) in the derivation and 0.848 (0.762–0.934) in the validation sample and, when including the treating hospital, the C-indexes were 0.914 (0.828–1.000) and 0.898 (0.810–0.986) in the derivation and validation samples, respectively.

**Table 4:** Predictors of mortality at 1 year in patients with colon cancer. Multivariate Cox regression analysis.

	Derivation sample			Weight	Validation sample		
	β (s.e.)	HR (CI 95%)	p-value		β (s.e.)	HR (CI 95%)	p-value
ASA (IV *vs*. I,II,III)	1.20 (0.40)	3.334 (1.509–7.367)	0.0029	**3**	1.55 (0.38)	4.693 (2.207–9.977)	<0.0001
Charlson index (≥4 *vs*. ≤4)	1.20 (0.29)	3.330 (1.884–5.886)	<0.0001	**3**	0.66 (0.28)	1.942 (1.123–3.358)	0.0175
Age (>75 *vs*. ≤75)	1.63 (0.29)	5.112 (2.893–9.035)	<0.0001	**4**	0.60 (0.28)	1.825 (1.055–3.157)	0.0315
pTNM							
• **III (*vs*. 0,I,II)**	-0.11 (0.41)	0.900 (0.403–2.011)	0.7976	**0**	0.67 (0.37)	1.955 (0.950–4.023)	0.0688
** **• **IV (*vs*. 0,I,II)**	1.52 (0.45)	4.566 (1.895–10.999)	0.0007	**4**	1.11 (0.44)	3.024 (1.288–7.100)	0.0110
Results of the surgery (R2 *vs*. R0-R1)	1.11 (0.40)	3.044 (1.381–6.709)	0.0058	**3**	2.06 (0.41)	7.817 (3.485–17.532)	<0.0001
Log Lymph nodes ratio							
** • -1.36< ≤-0.53 (*vs*. ≤-1.36)**	0.94 (0.46)	2.554 (1.035–6.298)	0.0418	**0**	-0.04 (0.46)	0.961 (0.393–2.351)	0.9312
** • -0.53< (*vs*. ≤-1.36)**	2.21 (0.38)	9.156 (4.325–19.381)	<0.0001	**5**	1.01 (0.35)	2.758 (1.399–5.439)	0.0034
C-index (95% CI)		0.865 (0.792–0.938)*				0.808 (0.734–0.882)†	
Adjuvant chemotherapy included							
ASA (IV *vs*. I,II,III)	1.42 (0.46)	4.130 (1.674–10.190)	0.0021	**3**	1.22 (0.46)	3.383 (1.378–8.303)	0.0078
Charlson index (≥4 *vs*. ≤4)	1.13 (0.36)	3.101 (1.524–6.310)	0.0018	**2**	0.57 (0.31)	1.767 (0.959–3.258)	0.0681
Age (>75 *vs*. ≤75)	1.44 (0.36)	4.216 (2.074–8.570)	<0.0001	**3**	0.64 (0.33)	1.906 (0.991–3.666)	0.0532
Adjuvant Chemotherapy and pTNM							
** **• **No and III (*vs*. Yes/No and 0,I,II)**	0.26 (0.56)	1.303 (0.434–3.914)	0.6371	**0**	1.55 (0.44)	4.702 (1.990–11.106)	0.0004
** **• **Yes and III (*vs*. Yes/No and 0,I,II)**	-0.01 (0.58)	0.989 (0.319–3.065)	0.9850	**0**	-0.28 (0.63)	0.759 (0.223–2.586)	0.6589
** **• **No and IV (*vs*. Yes/No and 0,I,II)**	1.92 (0.68)	6.829 (1.786–26.109)	0.0050	**4**	1.60 (0.66)	4.967 (1.359–18.155)	0.0154
** **• **Yes and IV (*vs*. Yes/No and 0,I,II)**	1.34 (0.63)	3.812 (1.103–13.168)	0.0344	**3**	1.09 (0.53)	2.965 (1.048–8.384)	0.0405
Results of the surgery (R2 *vs*. R0-R1)	1.37 (0.50)	3.950 (1.473–10.593)	0.0063	**3**	2.20 (0.44)	9.016 (3.838–21.181)	<0.0001
Log Lymph nodes ratio							
** **• **-1.36< ≤-0.53 (*vs*. ≤-1.36)**	1.23 (0.53)	3.421 (1.218–9.606)	0.0196	**0**	0.32 (0.50)	1.376 (0.519–3.649)	0.5210
** **• **-0.53< (*vs*. ≤-1.36)**	2.35 (0.46)	10.487 (4.249–25.880)	<0.0001	**5**	1.02 (0.40)	2.771 (1.259–6.096)	0.0113
C-index (95% CI)		0.885 (0.797–0.973)ⱡ				0.848 (0.762–0.934)§	

C-index: Concordance index. β: estimation. s.e.: standard error. HR: Hazard Ratio. CI: Confidence Interval. %: Percentage. *vs*.: versus.

Model without adjuvant chemotherapy included patients who died throughout the index hospital admission until one year after admission.

Model including adjuvant chemotherapy excluded patients who died throughout the index hospital admission or during the first 30 days after admission.

*Including the hospital (p = 0.9943), the c-index rise to 0.881 (0.807–0.955).

†Including the hospital (p = 0.8405), the c-index rise to 0.852 (0.776–0.928). In both case, statistically significant differences among some hospitals were found.

ⱡIncluding the hospital (p = 0.9141), the c-index rise to 0.914 (0.828–1.000).

§Including the hospital (p = 0.6881), the c-index rise to 0.898(0.810–0.986). In both case, statistically significant differences among some hospitals were found.

Predictors of mortality during the first 2 years after surgery were the same as those that predicted mortality during the first year (Table 5). The C-index showed a good discrimination for both samples (0.808 (0.751–0.865) and 0.796 (0.739–0.853) in the derivation and validation samples, respectively). When including the treating hospital in the model, the C-index rose to 0.832 (0.775–0.888) in the derivation sample and 0.819 (0.762–0.876) in the validation sample. When chemotherapy was included in the model C-indexes were 0.808 (0.749–0.867) in the derivation and 0.817 (0.760–0.874) in the validation sample and, when including the treating hospital, the C-indexes were 0.836 (0.777–0.895) and to 0.834 (0.775–0.893), respectively.

**Table 5:** Predictors of mortality at 2 year in patients with colon cancer. Multivariate Cox regression analysis.

	Derivation sample			Weight	Validation sample		
	β (s.e.)	HR (CI 95%)	p-value		β (s.e.)	HR (CI 95%)	p-value
ASA (IV *vs*. I,II,III)	0.80 (0.34)	2.223 (1.152–4.291)	0.0172	**2**	1.49 (0.32)	4.443 (2.389–8.263)	<0.0001
Charlson index (≥4 *vs*. ≤4)	0.73 (0.22)	2.077 (1.342–3.214)	0.0010	**2**	0.82 (0.22)	2.280 (1.492–3.484)	0.0001
Age (>75 *vs*. ≤75)	1.12 (0.21)	3.071 (2.017–4.675)	<0.0001	**3**	0.92 (0.21)	2.518 (1.653–3.835)	<0.0001
pTNM							
** **• **III (*vs*. 0,I,II)**	0.77 (0.29)	2.153 (1.229–3.774)	0.0074	**2**	0.77 (0.28)	2.151 (1.237–3.740)	0.0067
** **• **IV (*vs*. 0,I,II)**	2.07 (0.33)	7.932 (4.192–15.012)	<0.0001	**5**	1.51 (0.33)	4.546 (2.358–8.764)	<0.0001
Results of the surgery (R2 *vs*. R0-R1)	1.25 (0.31)	3.496 (1.901–6.428)	<0.0001	**3**	1.62 (0.38)	5.069 (2.430–10.574)	<0.0001
Log Lymph nodes ratio							
** • -1.36< ≤-0.53 (*vs*. ≤-1.36)**	0.50 (0.30)	1.655 (0.915–2.994)	0.0959	**0**	0.29 (0.32)	1.332 (0.707–2.511)	0.3747
** • -0.53< (*vs*. ≤-1.36)**	1.22 (0.27)	3.389 (1.979–5.803)	<0.0001	**3**	1.09 (0.28)	2.982 (1.739–5.113)	<0.0001
C-index (95% CI)		0.808 (0.751–0.865)*				0.796 (0.739–0.853)†	
Adjuvant chemotherapy included							
ASA (IV *vs*. I,II,III)	0.84 (0.34)	2.306 (1.175–4.525)	0.0152	**2**	1.31 (0.33)	3.711 (1.945–7.083)	<0.0001
Charlson index (≥4 *vs*. ≤4)	0.73 (0.23)	2.074 (1.318–3.264)	0.0016	**2**	0.78 (0.22)	2.173 (1.421–3.325)	0.0003
Age (>75 *vs*. ≤75)	1.14 (0.24)	3.130 (1.971–4.970)	<0.0001	**3**	0.74 (0.23)	2.093 (1.341–3.266)	0.0011
Adjuvant Chemotherapy and pTNM							
** **• **No and III (*vs*. Yes/No and 0,I,II)**	0.79 (0.36)	2.214 (1.099–4.459)	0.0261	**2**	1.36 (0.32)	3.904 (2.103–7.246)	<0.0001
** **• **Yes and III (*vs*. Yes/No and 0,I,II)**	0.79 (0.33)	2.196 (1.162–4.153)	0.0155	**2**	0.15 (0.35)	1.158 (0.583–2.299)	0.6756
** **• **No and IV (*vs*. Yes/No and 0,I,II)**	2.02 (0.49)	7.516 (2.875–19.651)	<0.0001	**5**	1.53 (0.50)	4.608 (1.740–12.199)	0.0021
** **• **Yes and IV (*vs*. Yes/No and 0,I,II)**	1.95 (0.37)	7.037 (3.389–14.612)	<0.0001	**5**	1.42 (0.38)	4.151 (1.983–8.689)	0.0002
Results of the surgery (R2 *vs*. R0-R1)	1.35 (0.34)	3.855 (1.988–7.476)	<0.0001	**4**	1.54 (0.38)	4.674 (2.226–9.815)	<0.0001
Log Lymph nodes ratio							
** **• **-1.36< ≤-0.53 (*vs*. ≤-1.36)**	0.58 (0.31)	1.787 (0.974–3.279)	0.0608	**0**	0.36 (0.33)	1.434 (0.758–2.714)	0.2681
** **• **-0.53< (*vs*. ≤-1.36)**	1.17 (0.29)	3.225 (1.820–5.715)	<0.0001	**3**	1.13 (0.29)	3.090 (1.765–5.409)	<0.0001
C-index (95% CI)		0.808 (0.749–0.867)‡				0.817 (0.760–0.874)§	

C-index: Concordance index. β: estimation. s.e.: standard error. HR: Hazard Ratio. CI: Confidence Interval. %: Percentage. *vs*.: versus.

Excluded patients who died throughout the index hospital admission or during the first 30 days after admission. Including patients who died during 1 year.

*Including the hospital (p = 0.4066), the c-index rise to 0.832 (0.775–0.888).

†Including the hospital (p = 0.2459), the c-index rise to 0.819 (0.762–0.876). In both case, statistically significant differences among some hospitals were found.

‡Including the hospital (p = 0.1713), the c-index rise to 0.836 (0.777–0.895).

§ Including the hospital (p = 0.3760), the c-index rise to 0.834 (0.775–0.893). In both case, statistically significant differences among some hospitals were found.

The predictive accuracy of each continuous and categorical risk scores for 1-year mortality and 2-year mortality were measured by the AUCs which are displayed in Table 6. The AUC of the continuous risk score for 1-year mortality was 0.876 (0.828–0.924) in the derivation sample and 0.780 (0.715–0.845) in the validation sample, without considering adjuvant chemotherapy. However, including adjuvant chemotherapy, the AUCs of the continuous risk score were 0.882 (0.826–0.939) and 0.791 (0.718–0.864) in the derivation and validation samples, respectively. A risk score was also developed for 2-year mortality. The AUCs were 0.806 (0.760–0.851) in the derivation sample and 0.807 (0.758–0.856) in the validation sample, without considering adjuvant chemotherapy. Including adjuvant chemotherapy, the AUCs of the continuous risk score were 0.804 (0.758–0.850) and 0.805 (0.756–0.855) in the derivation and validation samples, respectively. From the previous risk score, two categorical risk scores, with three categories each, were developed for both mortality periods (Table 6). The three risk categories of each risk score, for 1- or 2-year mortality, differentiated among the categories. The categorical risk scores developed showed AUCs of 0.854 (0.796–0.912) in the derivation sample and 0.766 (0.692–0.841) in the validation sample for 1-year mortality. For 2-year mortality, the AUCs for the derivation and validation sample were, respectively, 0.776 (0.725–0.826) and 0.779 (0.729–0.829), both including adjuvant chemotherapy. However, without considering adjuvant chemotherapy, the categorical risk scores developed showed AUCs of 0.838 (0.793–0.883) and 0.768 (0.708–0.827) in the derivation and validation samples, respectively, for 1-year mortality. For 2-year mortality, the AUCs for the derivation and validation samples were, respectively, 0.770 (0.722–0.818) and 0.778 (0.727–0.829). All the results were estimated without considering the effect of the treating hospital. As additional information, internal validation of all the models were performed where it can be seen that those variables selected for our 4 models are the ones more often obtained by bootstrap (Table 7).

**Table 6:** Categorical risk scores prediction of mortality at 1 or 2 years after surgery.

	Derivation sample			Validation sample		
	Total	Mortality	p-value	Total	Mortality	p-value
a. No Adjuvant Chemotherapy included						
*Mortality up to 1 year score*						
Risk score AUC (95% CI)	0.876 (0.828–0.924)			0.780 (0.715–0.845)		
Risk groups			<0.0001			<0.0001
** **• **0**	402 (44.92)	3 (0.75)		419 (46.50)	8 (1.91)	
** **• **1–5**	305 (34.08)	10 (3.28)		320 (35.52)	17 (5.31)	
** **• **≥6**	188 (21.01)	47 (25.00)		162 (17.98)	37 (22.84)	
AUC (95% CI)	0.838 (0.793–0.883)			0.768 (0.708–0.827)		
p Hosmer-Lemeshow test	1.0000			0.9999		
*Mortality up to 2 years score*						
Risk score AUC (95% CI)	0.806 (0.760–0.851)			0.807 (0.758–0.856)		
Risk groups			<0.0001			<0.0001
** **• **0–2**	464 (52.49)	16 (3.45)		481 (54.11)	18 (3.74)	
** **• **3–5**	305 (34.50)	37 (12.13)		313 (35.21)	32 (10.22)	
** **• **≥6**	115 (13.01)	47 (40.87)		95 (10.69)	48 (50.53)	
AUC (95% CI)	0.770 (0.722–0.818)			0.778 (0.727–0.829)		
p Hosmer-Lemeshow test	1.0000			1.0000		
b. Adjuvant Chemotherapy included						
*Mortality up to 1 year score*						
Risk score AUC (95% CI)	0.882 (0.826–0.939)			0.791 (0.718–0.864)		
Risk groups			<0.0001			<0.0001
** **• **0–2**	483 (55.07)	3 (0.62)		504 (56.88)	10 (1.98)	
** **• **3–6**	325 (37.06)	14 (4.31)		321 (36.23)	16 (4.98)	
** **• **≥7**	69 (7.87)	25 (36.23)		61 (6.88)	22 (36.07)	
AUC (95% CI)	0.854 (0.796–0.912)			0.766 (0.692–0.841)		
p Hosmer-Lemeshow test	1.0000			1.0000		
*Mortality up to 2 years score*						
Risk score AUC (95% CI)	0.804 (0.758–0.850)			0.805 (0.756–0.855)		
Risk groups			<0.0001			<0.0001
** **• **0–3**	551 (62.83)	20 (3.63)		594 (67.04)	23 (3.87)	
** **• **4–7**	263 (29.99)	39 (14.83)		237 (26.75)	40 (16.88)	
** **• **≥8**	63 (7.18)	34 (53.97)		55 (6.21)	33 (60.00)	
AUC (95% CI)	0.776 (0.725–0.826)			0.779 (0.729–0.829)		
p Hosmer-Lemeshow test	0.9999			0.9990		

AUC: Area Under the receiver operative Curve. %: Percentage, CI: Confidence Interval.

**Table 7:** Percentage of times each variable was selected by stepwise selection in internal validation using bootstrap.

	Predictors of mortality at 1 year in patients with colon cancer		Predictors of mortality at 2 years in patients with colon cancer	
	At index admission %	At 30 days (chemotherapy)%	At index admission %	At 30 days (chemotherapy)%
**Possible predictors**				
Age	**100**	**96**	**100**	**100**
Haemoglobin at baseline	72	18	59	44
Charlson index	**95**	**91**	**86**	**91**
ASA	**71**	**77**	**52**	**68**
Type of surgery	55	13	5	7
Organ invasion	9	0	6	3
Tumor sidedness	14	11	25	23
Histology	34	2	18	9
Result of the surgery	**73**	**76**	**92**	**93**
Log lymph node ratio	**100**	**100**	**96**	**95**
pTNM	**98**	—	**100**	—
Adjuvant Chemotherapy and pTNM	—	**91**	—	**100**

%: Percentage.

**Fig 2:**
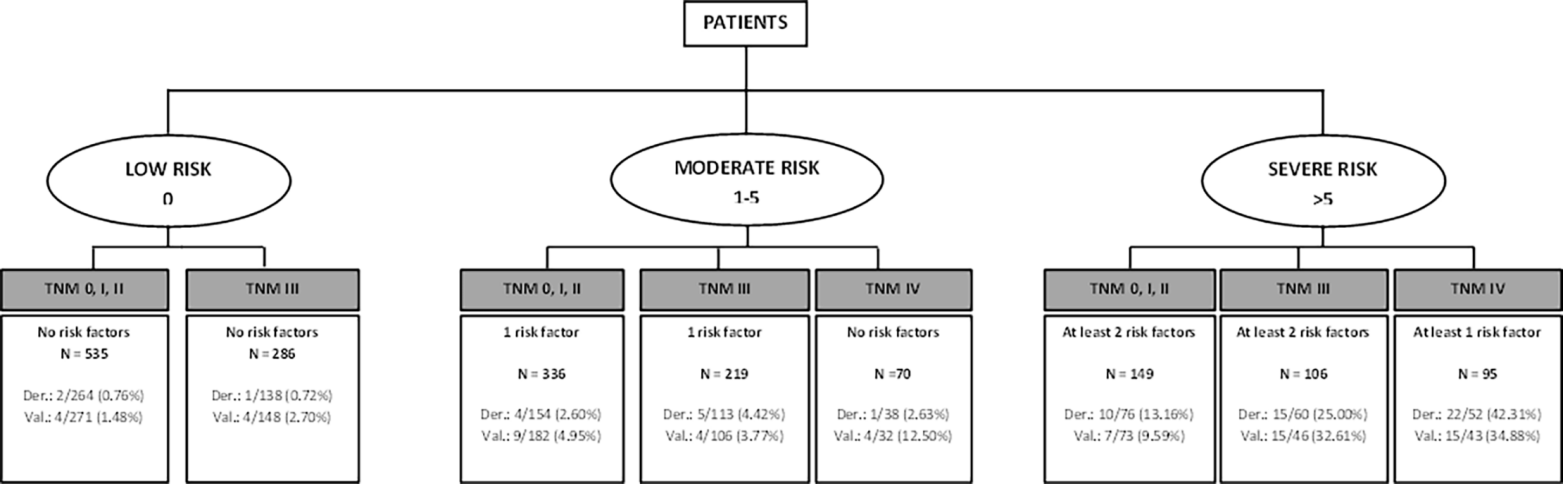
Detail of risk factors associated to one-year mortality risk categories. Risk factors include: ASA = IV, Charlson index ≥4, age>75 years, results of the surgery = R2 and Log lymph nodes ratio >-0.53.

**Fig 3:**
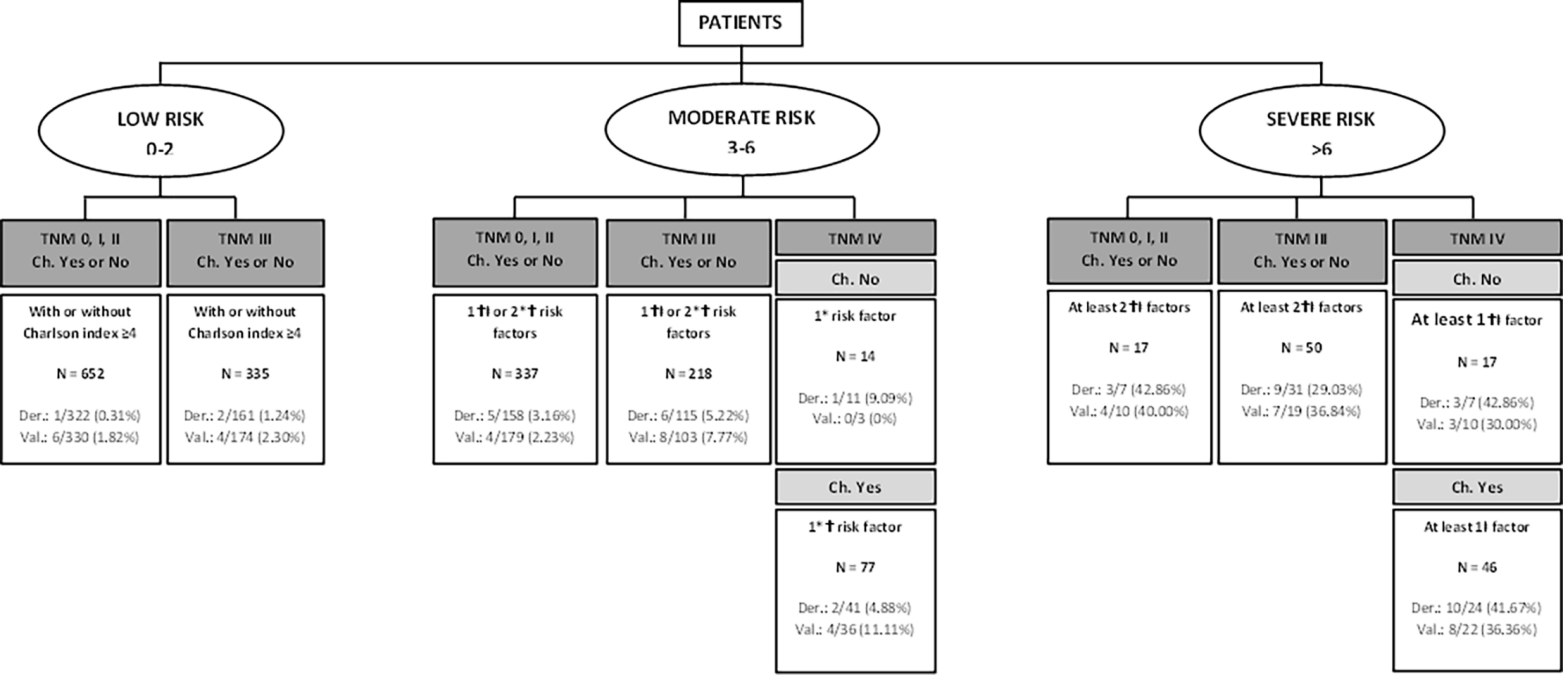
Detail of risk factors associated to one-year mortality risk categories, chemotherapy included. Risk factors include: ASA = IV, Charlson index ≥4, age>75 years, results of the surgery = R2 and Log lymph nodes ratio >-0.53.*Minor risk factor: Charlson index ≥4. †Moderate risk factors: ASA = IV, age>75 years and results of the surgery = R2. ⱡSevere risk factor: Log lymph nodes ratio >-0.53.

**Fig 4:**
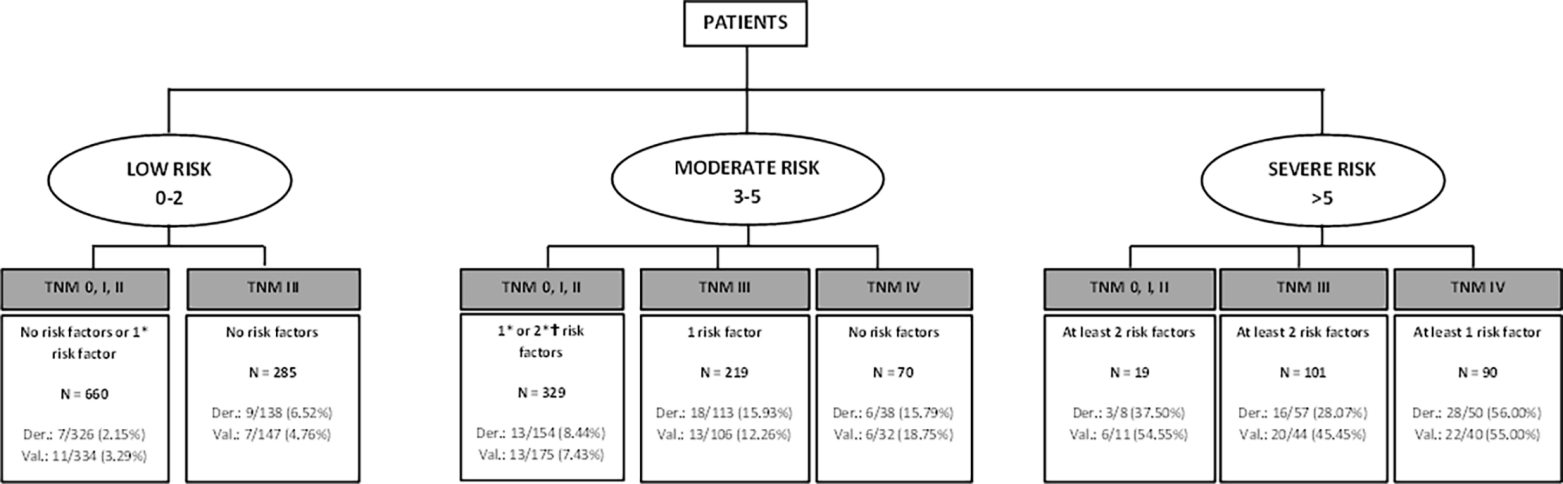
Detail of risk factors associated to two-years mortality risk categories. Risk factors include: ASA = IV, Charlson index ≥4, age>75 years, results of the surgery = R2 and Log lymph nodes ratio >-0.53.*Minor risk factor: Charlson index ≥4 and ASA = IV. †Moderate risk factors: age>75 years, results of the surgery = R2 and Log lymph nodes ratio >-0.53.

**Fig 5:**
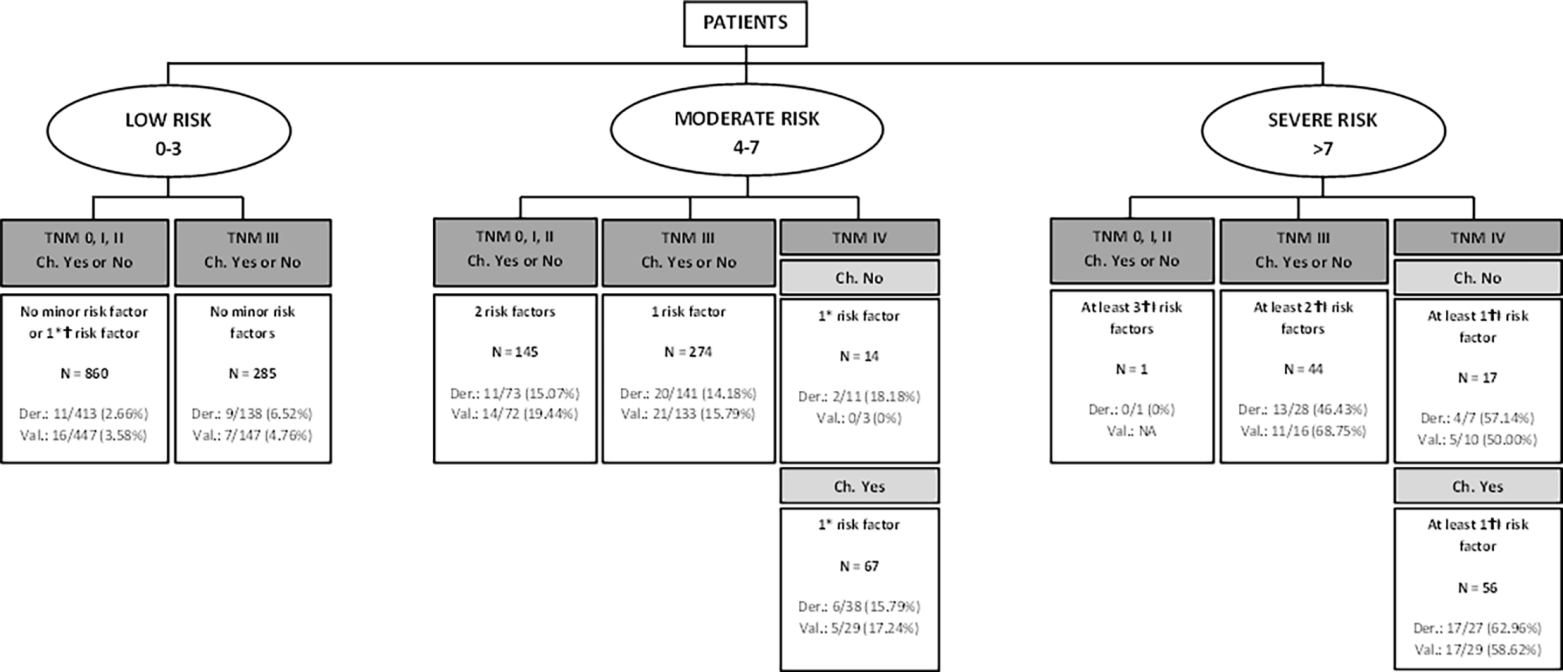
Detail of risk factors associated to two-year mortality risk categories, chemotherapy included. Risk factors include: ASA = IV, Charlson index ≥4, age>75 years, results of the surgery = R2 and Log lymph nodes ratio >-0.53.*Minor risk factor: Charlson index ≥4 and ASA = IV. †Moderate risk factors: age>75 years and Log lymph nodes ratio >-0.53. ⱡSevere risk factor: results of the surgery = R2. NA: Not applicable.

To better display the final results, trees charts derived from previous Tables 4 and 5 and the weights of the categories of the statistically significant variables and based on risk categories of Table 6 were developed. “Figs 2 and 3” presents the display for predicting mortality at one year with and without chemotherapy. “Figs 4 and 5” present similar trees but for two years mortality.

## Discussion

This prospective cohort study included a large sample of 1942 patients with colon cancer who underwent curative or palliative surgery. We identified factors before the intervention and up to 30 days and 1 year afterwards that related to mortality at 1 and 2 years in these patients. The categories of those factors were weighted to create continuous scores that, ultimately, were categorized into three categories of risk clinical prediction rules from minor to severe, based on the risk of dying at those points in time. Different prediction rules, though based in the same variables, were created for 1- and 2-year mortality, and, in each case, based on information available before surgery, during the index admission, and up to 30 days after surgery when, we took into account whether adjuvant chemotherapy was administered.[8–10, 18, 19]

We identified as predictors of medium-term mortality parameters such as ASA and comorbidities (based on the CCI). These are parameters related to the general condition of the patient before surgery, and are quite easily available for review. Similarly, older age can be related to higher frailty and need to be institutionalized or were more likely to undergo colon cancer resection during an unscheduled admission, which has been shown to be related to a higher rate of death in this patients. [20] We also identified TNM stage and lymph nodes ratio, which are related to the severity of the current disease, as a predictor of medium-term mortality. Finally, the residual tumor (R) classification, which is also related to the severity of the disease as well as reflects the result of surgery, had an influence on 1-year mortality. On the other hand, around 30 days after surgery is usually when, if it is indicated, the patient will begin chemotherapy for colon cancer. Adjuvant chemotherapy, as a specific treatment of the disease, has a clear relationship with mortality that changes depending on TNM stage. Therefore, a second set of prediction rules added chemotherapy treatment in conjunction with TNM stage to the previous parameters.

All of the predictors for 1-year mortality also appeared as predictors of 2-year mortality. These included those related to the general condition of the patient, such as age, ASA and CCI, but the main influence was from factors related to colon cancer and the patient’s evolution, including TNM stage. As with 1-year mortality, disease treatment, specifically adjuvant chemotherapy in relation to TNM stage, had a clear relationship with 2-year mortality.

Previous studies have found that disease severity, in this case, colon cancer stage as measured by TNM or lymph node status, or number of positive lymph nodes, or depth of primary tumor penetration are predictors of mortality.[21] Additionally, comorbidities, as measured by the Charlson comorbidity index in our case, jointly with age, have also been shown to affect mortality.[8, 20, 22, 23] Some studies have also implicated ASA level in mortality in colon cancer patients.[18, 24] Parameters from the evolution of disease in colon cancer patients, such as complications and reoperations, also were found to be predictive by some authors. Additionally, adjuvant chemotherapy was also a predictive factor in some studies, as in ours.[25] However, we first wanted to know which factors were predictors of mortality without considering chemotherapy. We introduced an interaction term to determine the different effects that chemotherapy had, depending on the TNM grade of the tumor. Additional variables, such as gender (a parameter that arose in some studies) and race, were not predictive in our analysis.[18, 26]

Most studies working on the identification of predictors of mortality in patients with colon cancer have focused on perioperative, in-hospital, or short-term mortality (up to 30 days post-operation). In this study, we explicitly removed this short-term mortality from our outcomes. We wanted to avoid the influence of surgeon ability, complications related to the surgery, and hospital health care quality in our prediction. However, as we have shown in our results, the variable of the treating hospital did have an influence on the 1- and 2-year mortality, and we have adjusted for it in our models. This means that the specific health care location has a role in explaining the causes of mortality, which indicates a possible problem of variability and equity among our centers.[26, 27]

From a practical point of view, our summary trees are developed to have a quick and simultaneous view of the prognosis of a patient at one and two years after surgery just at the time of the index admission-without chemotherapy- and, additionally, at 30 days afterwards, when chemotherapy is added. In general terms, mortality is low and remains similar at 1 an 2 years for TNM 0/I/II with no risk factors or just the Charlson comorbidity index ≥4. On the opposite side, is high for TNM IV or III with additional risk factors based on our rules. Those graphics can help clinicians as well as patients.

This study has several strengths. It was based on a large prospective cohort obtained from 22 hospitals, thus providing variability. The number of variables on the possible predictors of death was quite large. We have also included models with and without chemotherapy. The development of the predictive models followed the structure of current guidelines, such as those included in the TRIPOD statement [28], including an internal and external validation of all models. We identified predictive factors common to various other studies. However, the strength of our study lies in the fact that we have put them together, with just a few variables that are usually recorded and easily accessed, into single scores that may help clinicians easily and quickly classify the prognosis of these patients. As our scores have excellent predictive ability, clinicians will be better able to guide patients in follow-up. These scores may also serve as a reference to patients and families.

However, this study also has limitations. As in any prospective multicenter study with a 2-year follow-up, loss to follow-up was our main source of bias. Though reviewers in each center were trained to collect all of the planned information from all of the patients recruited at baseline, loss of patients and information was inevitable, though contained. We did study the influence of various HRQoL parameters in our outcomes, but, surprisingly, none of the questionnaires studied, even those detailing markers of frailty or health status, showed any predictive influence on mortality. Additionally, none of the tumor biomarkers studied, such as carcinoembryonic antigen levels, showed any relationship with mortality. Nevertheless, as new genetic and biological markers are discovered, they should be considered for addition to our models to increase predictive ability. Even further, improvements in the treatment of these patients, as the addition of new and more effective drugs, may change the predictive ability of our models though more than 50% of our patients did not receive chemotherapy and just those more severe received the new molecular targeted agents. We also did not do in-depth analysis on the influence of the quality, appropriateness, or existence of errors in the health care to the patients, or adherence to various treatments for the disease and comorbidities, when all of these factors could have a clear influence on mortality.

Finally, there are various statistical techniques currently used to develop clinical prediction rules. The ones we used in our study, namely, survival and logistic regression models, are classical but robust and clear for reading comprehension. Other techniques that have been employed in other studies, such as neural networks or a Bayesian belief network, may be alternatives, though some of them have a “black box” effect, wherein the design of the model is obscure to the reader. They are also usually no superior to the classical techniques.[10, 18, 29] As with any clinical prediction rule, proper external validation of our proposed models is the necessary next step, and, from a practical clinical point of view, studies are needed where the use of these rules improve patient health care.[30]

In conclusion, these clinical prediction rules provide clinicians and patients with decision tools that, with a minimal amount of easily obtainable information, have shown good predictive abilities and can be used in the clinical decision process. They provide information before starting the chemotherapy treatment process and are also valuable tools once the chemotherapy treatment has started in those patients where it has been prescribed since we present models for both situations. Lastly, future studies should examine the validity of our models in other settings, as well as apply them in real-life situations to determine their usefulness in the patient care and management process.
